# Sensorimotor memories influence movement kinematics but not associated tactile processing

**DOI:** 10.1038/s41598-023-45138-8

**Published:** 2023-10-20

**Authors:** Marie C. Beyvers, Dimitris Voudouris, Katja Fiehler

**Affiliations:** 1 Department of Experimental Psychology, Justus Liebig University Giessen, Otto-Behaghel-Strasse 10F, 35394 Giessen, Germany; 2 Center for Mind, Brain and Behavior (CMMB), University of Marburg and Justus Liebig University Giessen, Giessen, Germany

**Keywords:** Psychology, Human behaviour

## Abstract

When interacting with objects, we often rely on visual information. However, vision is not always the most reliable sense for determining relevant object properties. For example, when the mass distribution of an object cannot be inferred visually, humans may rely on predictions about the object’s dynamics. Such predictions may not only influence motor behavior but also associated processing of movement-related afferent information, leading to reduced tactile sensitivity during movement. We examined whether predictions based on sensorimotor memories influence grasping kinematics and associated tactile processing. Participants lifted an object of unknown mass distribution and reported whether they detected a tactile stimulus on their grasping hand during the lift. In Experiment 1, the mass distribution could change from trial to trial, whereas in Experiment 2, we intermingled longer with shorter parts of constant and variable mass distributions, while also providing implicit or explicit information about the trial structure. In both experiments, participants grasped the object by predictively choosing contact points that would compensate the mass distribution experienced in the previous trial. Tactile suppression during movement, however, was invariant across conditions. These results suggest that predictions based on sensorimotor memories can influence movement kinematics but not associated tactile perception.

## Introduction

Successful human-world interactions require processing of relevant sensory signals. For example, when grasping an object, humans choose grasping points based on visual information about the object’s size^1^, orientation^2^, shape^3^, or surface material^4^. However, vision is not always the most reliable sense to work out relevant object properties. Indeed, although object mass can be inferred from visual information, as mass typically increases with object size, there are occasions when vision misleads our judgements, such as the size-weight illusion^5^. Likewise, although an object’s mass distribution can be inferred by the object’s shape, some objects that appear to be symmetric may actually have an asymmetric mass distribution. When grasping objects with properties that cannot be reliably inferred from vision, such as of unknown mass distribution, humans *appear* to adopt a generic grasping behavior^6^. If the mass distribution remains invariant over repeated trials, humans rapidly learn and establish more reliable predictions about the object’s properties, which helps to tailor their motor behavior by adopting more suitable grasping postures and by applying more efficient digit forces that foster a stable grasp and object manipulation^7^. In such cases, it is evident that humans learn the object dynamics through prior experiences that arise from somatosensory feedback during object interactions.

Despite the importance of somatosensory feedback for motor control and learning, somatosensory afferents, in particular those from the tactile domain, are typically suppressed during movement^8–13^. This phenomenon of tactile suppression is primarily explained by an internal feed-forward model that predicts future sensory states of the moving limb and suppresses associated sensory signals^14, 15^ based on efferent signals related to the underlying movement^16–18^. Tactile suppression is typically assessed by presenting tactile stimuli on a limb when it is moving compared to resting. It has therefore been suggested that tactile suppression may not be sensitive only to sensorimotor predictions, but also to other factors, such as backward masking or attentional processes due to dual-tasking. For instance, peripheral mechanisms, such as proprioceptive afferents arising from the movement itself may obstruct the perception of the tactile stimulus on the moving limb through backward masking^19^. However, tactile suppression is not stronger at moments when sensory feedback signals are stronger, such as when grip and load forces are increased^20^. Tactile suppression is also robust to increased cognitive demands when performing more than one task^22^, and it occurs only when the probing tactile stimuli are presented on the moving limb, and not on a static limb while another limb is moving^12^, further supporting the idea that suppression does not simply occur due to *any* type of movement. Instead, tactile suppression is a process related to sensorimotor predictions^14^, reflecting the interplay between predictive and sensory feedback signals^6, 21^.

Sensorimotor predictions are based on a combination of current sensory feedback and prior knowledge about the prevailing dynamics^23^. When statistical regularities are highly systematic, such as when repeatedly grasping an object of constant mass distribution, sensorimotor predictions are used both to adopt a suitable grasping posture and to suppress associated tactile feedback^6^. Likewise, when the object’s mass distribution changes on a trial-by-trial basis in an unpredictable manner, the adopted grasping configuration remains similar and the associated tactile suppression is substantially weaker^6^. However, even in such unpredictable environments, humans plan their movements on the basis of predictions inferred through their most recent sensorimotor memories^24, 25^. Specifically, people grasp an object of unknown mass distribution *as if it had* the mass distribution experienced in the previous trial. Thus, predictive control seems to be preserved even when acting within unpredictable worlds, and even if these predictions may eventually be incorrect.

In the present study, we sought to examine whether such predictions based on sensorimotor memories influence grasping kinematics and associated tactile processing. We asked participants to grasp and lift an object with a mass distribution that could not be inferred from visual information. The object’s mass distribution changed in a pseudo-randomized order across trials but, unbeknownst to the participants, consecutive trials could involve either the same or different mass distributions. At the moment of object contact, a brief vibrotactile probe stimulus on the index finger of the grasping hand was presented to probe tactile suppression^6, 10^ and participants had to report whether they detected that probe stimulus or not. We probed tactile suppression right at the moment of contact, and not later, to isolate the effects of the sensorimotor prediction, without contamination from subsequent sensory feedback that could either confirm or contradict this prediction. This time of probing is used to assess participants’ reliance on predictive and feedback processes, with stronger suppression reflecting weaker reliance on feedback signals and a stronger reliance on predictive processes^6, 14^. Specifically, when reliance on sensory feedback is reduced, for instance because participants rely more on predictive signals, then sensory sampling from the moving limb will be downweighed, leading to the suppression of the probe tactile stimulus. Likewise, in cases where sensory sampling is more important, the associated sensory feedback from the moving limb will be prioritized, and therefore the probing tactile stimulus on the moving hand will be less suppressed or even enhanced.

Based on previous work^24^, we expected previous trial effects on various kinematic variables. We also expected tactile suppression during grasping compared to rest^6, 10^. If tactile suppression is modulated by predictions established on the basis of sensorimotor memories, we expected stronger tactile suppression when the mass distribution of an object is repeated from one trial to another compared to when it changes.

## Experiment 1

### Methods

#### Participants

Twenty-four participants (18 female, 6 male) aged 19–34 years (*mean* = 23 ± 3.6) participated in this experiment. In the context of tactile suppression, our study is the first to examine sequence effects. Thus, no data were available on the basis of which we could have calculated an a priori power analysis. Therefore, our sample size was based on previous studies on tactile suppression^6, 8, 11, 14, 22, 26^. Participants were all right-handed as measured by the German translation of the Edinburgh Handedness Inventory^27^ (*range* = 60–100), free from any known neurological conditions, and had normal or corrected-to-normal visual acuity. All participants gave informed written consent to participate in the experiment and received 8 €/hour or corresponding course credits as compensation for their effort. The experiment was approved by the research ethics board of the Department of Psychology and Sports Science, Justus Liebig University Giessen and was in accordance with the Declaration of Helsinki (2013, except pre-registration of the study).

#### Apparatus

**Figure 1:**
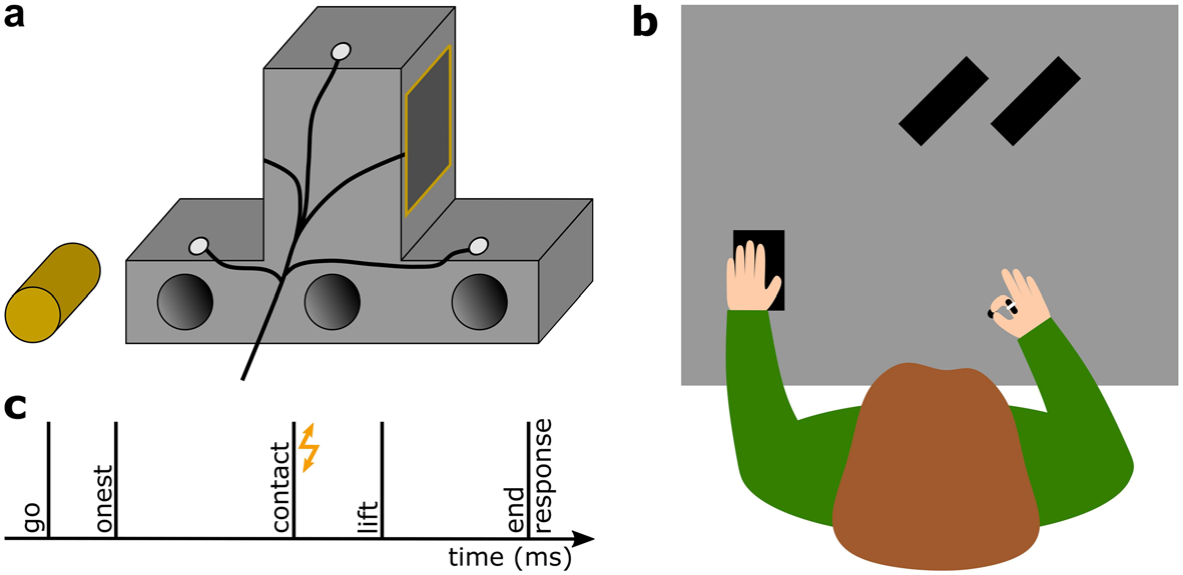
Experimental setup. (**a**) Illustration of the backside of the inverted T-shaped object with three infrared markers (white circles) and touch sensors (here only one visible) fixed to the grasping area of the object. The three tubes in the object’s base were never visible to the participant. The brass mass is depicted to the left of the object. (**b**) Top view of the setup with the right index finger and thumb resting on the start position and the left hand resting on the numpad. A vibrotactile sensor was fixed on the dorsal part of the participant’s right index finger. Both object positions are also depicted (black rectangles). (**c**) Timeline of a single trial with the flash illustrating the vibrotactile stimulation at the moment of object contact.

Participants had to grasp and lift an inverted T-shaped object (Fig. 1a). The object had a depth of 5 cm. The lower part was 15 cm wide and 3 cm high, while the upper part was 5 cm wide and 7 cm high. At the backside of the lower part of the object, invisible to the participants, three tubes were distributed laterally. A cylindrical piece of brass (116 g) was inserted in either the left or the right tube, creating an asymmetric object’s mass distribution (MD). The total mass of the object, including the brass, was 273 g.

Participants were seated comfortably in front of a 117 cm × 80 cm table (Fig. 1b). A small keypad (12.5 cm × 8 cm) on the table was under the participant’s left hand at a comfortable position. The start position for each reaching-to-grasp movement was aligned to the participants’ right shoulder and was marked by a round piece of felt pad located ~ 30 cm in front of their body. To avoid stereotyped movements, two different lateral object positions were used, each 34 cm from the start position. To prevent participants from seeing in which tube the experimenter inserted the brass prior to each trial, participants wore liquid–crystal shutter glasses (PLATO, Translucent Technologies, Toronto, Canada) throughout the grasping block (see below).

To track the movement of the participants’ hand and of the object, the position of five infrared markers were recorded at 100 Hz with an Optotrak Certus motion tracking system (Northern Digital Inc., Waterloo, ON, Canada). One marker each was attached to the fingernail of the participants’ right thumb and index finger. The other three markers were attached in a triangular arrangement on the backside of the object.

Vibrotactile stimulations, always given at the moment of object touch, were triggered by a touch sensor (4.37 cm × 4.37 cm; Interlink Electronics Inc., Westlake Village, CA, USA) that was mounted on each grasping side of the upper part of the object and that was connected through a NI USB-6009 device (National Instruments Corporation, Austin, TX, USA) to the host PC. A vibrotactile stimulation device (Engineering Acoustics Inc., Casselberry, FL, USA) was attached to the dorsal part of the medial phalanx of the participants’ right index finger. To allow for a natural grasp, we did not cover the fingertip of the participants with the tactor but chose to stimulate close to the site critical for the movement, as tactile suppression has a rather low spatial specificity across the hand^19^. The presented vibrotactile stimuli (250 Hz, 50 ms) differed in intensity; six stimuli had a peak-to-peak displacement ranging from 9.4 to 56.7 µm in steps of 9.4 µm, while one third of the trials involved no stimulation. As tactile information becomes increasingly relevant the closer the hand moves to an objects’ surface^11^, we applied tactile stimulation at the moment of first object touch, just before participants lifted the object and received sensory information about its mass distribution. This enabled us to pick up the original prediction regarding object dynamics and examine its impact on tactile processing. This is also in line with previous studies^6, 20^ showing that the timepoint when participants made first contact with the object is sensitive to predictive tactile suppression.

Care was taken to ensure that motion tracking markers, the tactor and the connected cables did not constrain the participants’ freedom of movement.

#### Procedure

The experiment consisted of three blocks: two baseline blocks and one grasping block. In the baseline blocks, participants rested their right hand in a comfortable position in front of them. Each baseline trial started automatically: a probe vibrotactile stimulus was presented, if at all, 500 ms after the beginning of the trial. An auditory cue 500 ms after that moment prompted participants to press a button underneath their left index finger or thumb and indicate whether they felt a vibration or not, respectively. The next trial started automatically 500 ms after the response was given. During each of the two baseline blocks, each of the six stimulus intensities was presented five times, while the no-stimulation catch trials were presented 15 times, resulting in a total of 45 trials presented in a pseudorandom order. One baseline block each was presented before and after the grasping block in order to account for possible changes in tactile sensitivity over time.

During the grasping block, each trial started with the shutter glasses being opaque and the participant bringing their right thumb and index fingertips together at the start position. The experimenter inserted the brass mass into the appropriate tube and then placed the object on the appropriate position. Even if the mass distribution was similar between two consecutive trials, the brass was always pulled out and inserted into the given tube to keep auditory cues constant. After the experimenter pressed a key to start the trial, the shutter glasses turned transparent and an auditory signal indicated that participants could start their movement. They were to reach out and grasp the object with their right thumb and index finger at both sides of its upper part, where the touch sensors were attached, lift it as straight as possible for ~ 10 cm, before placing it back down on the table and returning their hand to the start position. Once participants first touched the object, as this was detected by the first activation of the touch sensors, a probe vibrotactile stimulus could be presented on the participant’s right index finger. These probe stimuli were identical to those presented in the baseline blocks. After five seconds following the auditory signal, data collection was stopped, the shutter glasses turned opaque, and an auditory cue prompted participants to indicate whether they had felt a probe stimulus (yes/no response). Participants responded in the same way as in the previously described baseline procedure. Figure 1c illustrates the progression of a single grasping trial.

In the grasping block, each participant was presented with a trial sequence of mixed mass distributions. This was designed in a way that all possible combinations of consecutive mass distributions occurred equally often during the block: left followed by left (LL), right followed by left (RL), left followed by right (LR), and right followed by right (RR). For each of these four combinations, 30 trials with vibrotactile stimuli and 15 without a stimulation were presented, identical to those presented in one baseline block. The sequence was designed so that there would appear to be no regularity in the sequence of combinations, either in the mass distribution or in the intensities of the tactile stimuli. This mixed sequence consisted of trials where the mass distribution repeated (*mixed*_*same*_; LL and RR) or changed (*mixed*_*different*_; LR and RL) from one trial to the next. Overall a total of 180 trials were presented, with 90 trials having the object on the left and the other 90 trials on the right target position in a pseudorandom order across trials. Before starting the grasping block, participants performed nine practice trials to familiarize with the task and the object weight. For those trials, the brass cylinder was placed into the central tube of the object, and this was the only case when the symmetric mass distribution was used. In these practice trials, each stimulus intensity was presented once, in addition to three catch trials with no stimulation. The object was randomly placed five times on the left position and four times on the right position, or the other way around, counterbalanced across participant.

#### Data analysis

The first goal of the study was to examine whether participants plan their upcoming movement on the basis of sensorimotor memories, i.e. sensorimotor information experienced in the previous trial. To analyze the relevant kinematic behavior, we examined the influence of the previous trial’s MD on (a) the vertical separation of the digits at the moment of first object contact, (b) the time between first object contact and object lift, and (c) the maximal object roll during lifting. To determine the relevant kinematic measures for each trial, we first calculated the speed of the hand and of the object by numerical differentiation of the mean position of the markers on both digits and on the object, respectively. These two speed vectors were dual-pass filtered using the MATLAB *filtfilt* function with a 2nd order lowpass butterworth filter and a cutoff frequency of 0.3 Hz. Object lift onset was defined as the first timepoint at which the object speed was greater than 10 cm/s. The moment of object contact was determined as the last timepoint before object lift onset with a hand speed lower than 10 cm/s. For each trial, we calculated the *digits’ separation* as the mean vertical distance between the thumb and the index finger within the first 100 ms from the moment of object contact, with positive values indicating that the thumb was placed higher than the index finger^6^. *Loading time* was defined as the time between the moment of contact and object lift onset. Finally, *object roll* was defined as the absolute maximal tilt angle within the first 250 ms after the onset of the object lift.

**Figure 2:**
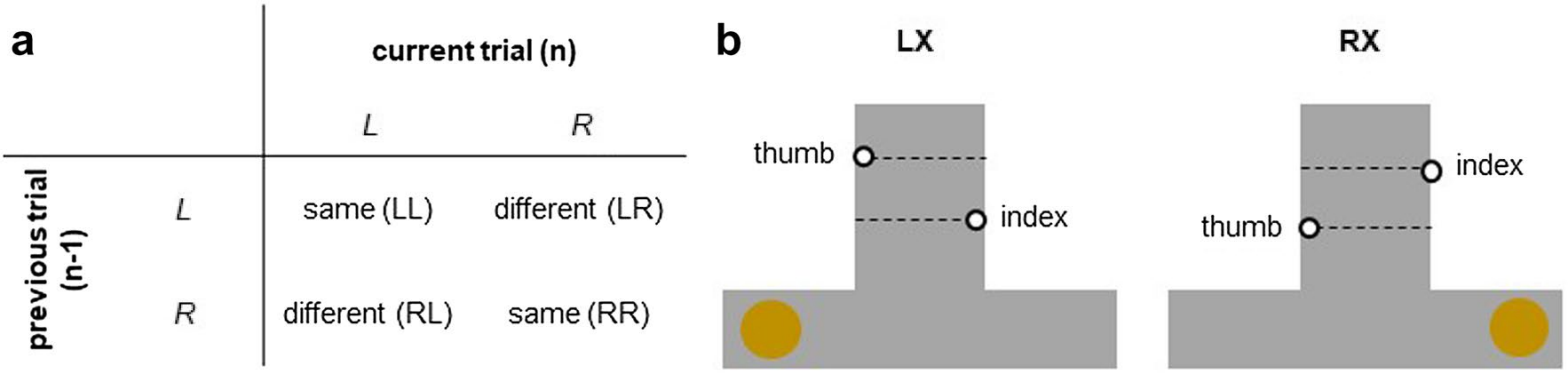
Previous trial configurations. (**a**) Possible combinations of previous and current trial MDs. The *MD*_*same*_ configuration involved repeats of the same MD in two consecutive trials (LL & RR), whereas the *MD*_*different*_ configuration involved a change of MD (RL & LR). (**b**) Expected positioning of the digits on the object, based on previously experienced MD. The previous position of the brass mass is represented by a golden circle. With a previous MD on the left (LX), a higher placement of the thumb was expected in the current trial X, and with a previous MD on the right (RX), a higher placement of the index finger was expected in the current trial X.

Next, we examined whether kinematic behavior on a trial with a given MD was influenced by the MD experienced in the previous trial. We defined two configurations of consecutive MDs: *MD*_*same*_ involved consecutive MDs that were identical (LL, RR), whereas *MD*_*different*_ involved different consecutive MDs (RL, LR). Overall, each trial (n) was classified based on the mass distribution presented in the preceding trial (n − 1). For instance, if the MD in the first four trials of a block was RRLL, we classified the second trial (R) as ‘*MD*_*same*_’, the third trial (L) as ‘*MD*_*different*_’, and the fourth trial (L) as ‘*MD*_*same*_’ (see also Fig. 2a). For each trial, we calculated the *digits’ separation* as the difference in the vertical position between the markers on the thumb and index fingers at the moment of object contact, with positive values indicating that the thumb was positioned higher than the index finger. Please note that each trial, except the first of the block, was both the first and the second trial of a possible pair, and that only the second trial of each pair was used to calculate the digits’ separation. After calculating this for all trials, we averaged the values across the four conditions (LL, RR, LR, RL) to obtain four average vertical distance values per participant. To avoid negating the actual effect, the average digits’ separation in trials with left MD were subtracted from the corresponding value in trials with right MD, separately for *MD*_*same*_ (RR–LL) and *MD*_*different*_ (LR–RL) configurations. Based on previous work^6, 24^, we expected participants to place their thumb lower and higher than the index finger when they anticipated the mass to be on the right and left side, respectively, which would minimize object roll during object lift (see Fig. 2b). As these would lead to negative and positive digits’ separation indices (see above), respectively, the *MD*_*same*_ calculation (RR–LL) should yield negative indices and the *MD*_*different*_ calculation (LR–RL) should yield positive indices. In other words, if the resulting index is negative when the mass distribution is repeated and positive when the mass distribution is switched, this should indicate that participants use predictive control and adopt in trial *n* a grasp configuration to match the dynamics experienced in trial *n* − 1, independently of whether the adopted grasping posture is suitable for the mass distribution of the current object. *Loading time* and *maximal object roll* were calculated based on the average values that were obtained for each trial for both configurations (*MD*_*same,*_* MD*_*different*_).

The second goal of the study was to examine whether sensorimotor memories influence somatosensory processing on the grasping hand. In accordance with previous work^6, 10^, we expected decreased sensitivity to the probing vibrotactile stimuli during grasping compared to the resting trials. If participants consider the MD experienced in the previous trial in order to predict the MD in the upcoming trial, tactile sensitivity should change accordingly: tactile suppression should be stronger in the second of two consecutive trials with identical MD, as the predicted and experienced movement dynamics match. Alternatively, if tactile suppression is affected by backward masking, we expect stronger suppression in trials with different MD than predicted, due to increased feedback processing^28^.To assess tactile suppression, we first merged the responses of both baseline blocks and fitted each participant’s responses in each condition (baseline, *MD*_*same*_, *MD*_*different*_) with separate logistic functions using maximum-likelihood estimation with the function *psignifit 3*^29^ in MATLAB 2019b (MathWorks, Natick, MA). For each of these three psychometric functions that we obtained for each participant, we estimated the detection threshold as the stimulus intensity at 50% of the function. To quantify tactile suppression during grasping while accounting for individual tactile sensitivity, each participant’s baseline detection threshold was subtracted from their respective values of the two grasping configurations (*MD*_*same*_, *MD*_*different*_). Each of these suppression values were calculated for each participant, and then averaged across participants, with higher positive values indicating stronger tactile suppression. Participants only took part in the whole experiment if their false positive rate in the baseline trials was below 30%. In the grasping block, false alarm rates were below 30% for all participants. Further, we merged the two baseline blocks because we found no differences in the respective detection thresholds.

After calculating the above-mentioned variables, we examined effects of sensorimotor memories on grasping kinematics and associated tactile suppression. For kinematic behavior, we submitted the index values for digits’ separation to two-sided one sample t-tests against zero, which tested whether participants adopted a grasping posture based on the mass distribution experienced in the trial before. For tactile processing, we examined whether detection thresholds during each grasping condition was greater than those during baseline by submitting the suppression scores in two-sided one sample t-tests against zero. In a second level, we investigated the effect of previous mass distribution on all kinematic variables and tactile suppression, by using separate paired samples t-tests for grasping kinematics and tactile processing between trials with repeating (*MD*_*same*_) and changing MD (*MD*_*different*_). Effect sizes are described as Cohen’s d. All statistical analyses were carried out with JASP (Version 0.14.1) and plots were generated using R (Version 1.4.1717) and the package ggplot2 (Version 3.3.5).

### Results

#### Kinematics

**Figure 3:**
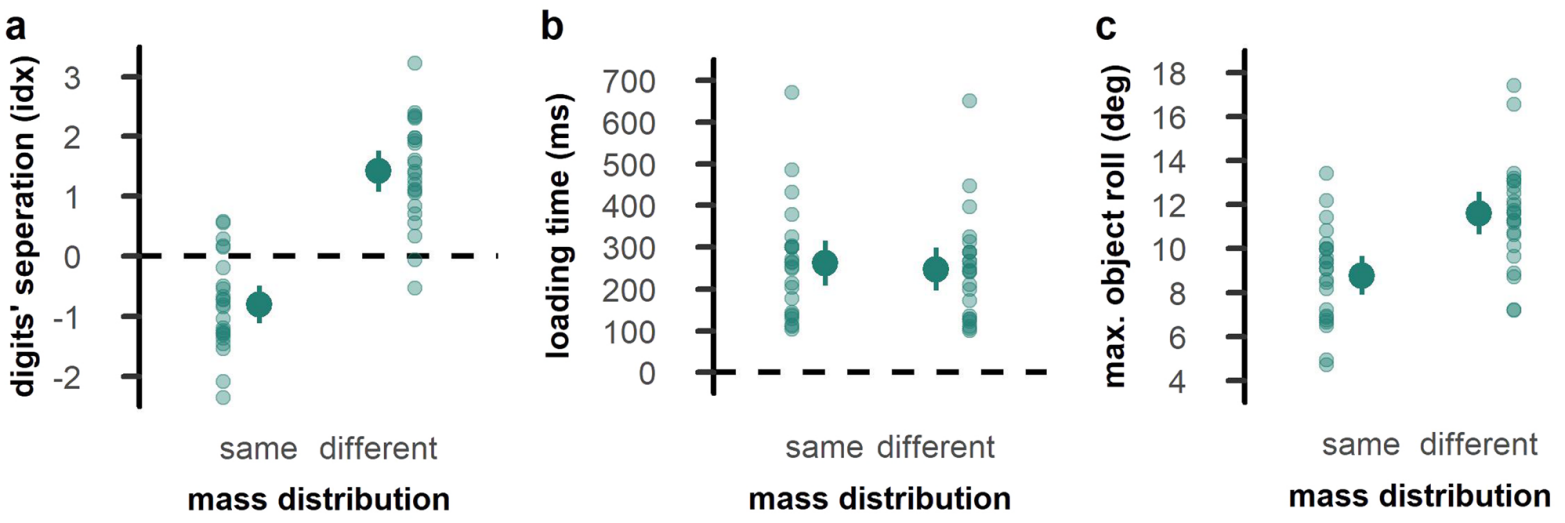
Kinematic performance in Experiment 1. (**a**) Digits’ vertical separation at the moment of object contact, (**b**) loading time, and (**c**) maximal object roll during the first 250 ms after object lift. The left side of each panel shows trials with a repeated mass distribution, and the right side for trials with a changing mass distribution. Opaque dots represent the mean across participants with error bars indicating the confidence interval. Transparent dots represent individual participants.

Regarding finger positioning on the object, we found mean index values below zero for the *MD*_*same*_ condition (*t*_*23*_ = − 5.07, *p* < 0.001, *d* = − 1.04) and above zero for the *MD*_*different*_ condition (*t*_*23*_ = 8.08, *p* < 0.001, *d* = 1.65), indicating that participants positioned their fingers in anticipation of a MD that was identical to the one presented in the trial before, independently of whether this prediction was correct or not. Accordingly, the index value was smaller for *MD*_*same*_ than *MD*_*different*_ (*t*_*23*_ = − 10.83, *p* < 0.001, *d* = − 2.21; Fig. 3a).

The MD experienced in the previous trial also affected loading time (*t*_*23*_ = 5.42, *p* < 0.001, *d* = 1.11; Fig. 3b) and maximal object roll (*t*_*23*_ = − 18.93, *p* < 0.001, *d* = − 3.87; Fig. 3c) in the current trial. Specifically, loading times were longer and object roll was smaller when the MD was repeated from one trial to another compared to when it was changed.

#### Tactile sensitivity

**Figure 4:**
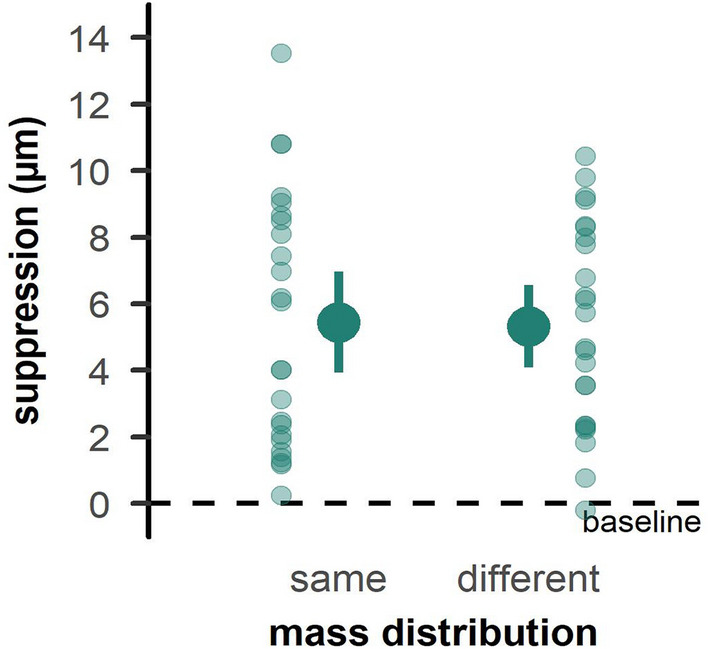
Tactile suppression scores in Experiment 1. The left side of each panel shows trials with a repeated mass distribution, and the right side trials with a changing mass distribution. Zero values indicate no change from baseline, while higher values indicate a deterioration of tactile perception. Details as in Fig. 2.

As expected^6, 10^, tactile suppression was evident during grasping compared to rest. This was the case for both the *MD*_*same*_ (*t*_*23*_ = 7.04, *p* < 0.001, *d* = 1.44) and *MD*_*different*_ (*t*_*23*_ = 8.452, *p* < 0.001, *d* = 1.72). Yet, we found no effects of previous trial MD on tactile suppression (*t*_*23*_ = 0.33, *p* = 0.742, *d* = 0.07; Fig. 4).

### Discussion Experiment 1

We investigated whether and how sensorimotor memories influence grasping kinematics and associated tactile processing. Our results show that, when grasping an object with uncertain properties, humans utilize sensorimotor memories based on previous trial experience to plan their next movement^24^. Specifically, participants grasped an object of unknown mass distribution by predictively choosing contact points that would compensate the mass distribution experienced in the *previous* trial, even if the mass distribution in the current trial was different than predicted. These results suggest that sensorimotor memory is used to adjust movement kinematics when object properties are uncertain and sensory action consequences are hard to predict. In line with previous findings^6, 10^, tactile sensitivity was decreased during grasping compared rest reflecting movement-induced tactile suppression. However, tactile suppression was unaffected by the mass distribution of the previous trial, as it did not differ between consecutive trials of repeating and changing mass distributions. These results suggest that tactile suppression is neither affected by predictions based on sensorimotor memories (*MD*_*same*_ > *MD*_*different*_), nor by backward masking (*MD*_*same*_ < *MD*_*different*_).

In this experiment, participants had no information about the mass distribution as this could change from trial to trial. We also probed tactile suppression at a moment when the current mass distribution could not be confirmed. Thus, any predictions based on sensorimotor memories might have been equally strong for trials that involved the same (*MD*_*same*_) compared to trials that involved a different mass distribution than the one predicted (*MD*_*different*_). To address this possibility, we conducted Experiment 2, where we interleaved longer sequences of trials involving an object of constant mass distribution with shorter sequences of trials with changing mass distribution. We assumed stronger sensorimotor predictions in sequences of constant compared to mixed distributions, and thus expected stronger suppression in those sequences. This experiment was further split into two sessions: in the first session, participants were exposed to the interleaved constant and mixed sequences to test for implicit learning of the statistical regularities of the trial sequence. In the second session, the experimenter informed the participants explicitly about the upcoming sequence (constant or mixed). This was done to explore whether explicit information about the upcoming trial sequence would alter predictive control and the strength of tactile suppression with stronger suppression for the constant compared to the mixed sequence.

## Experiment 2

### Methods

#### Participants and apparatus

Twenty-four participants (14 female, 10 male) aged 18–31 years (*mean* = 22.71 ± 3.46) participated in the study and completed the experiment. None of the participants took part in Experiment 1. They were all right-handed as measured by the Edinburgh handedness inventory (range = 60–100) and had normal or corrected-to-normal visual acuity. As in Experiment 1, participants gave their written informed consent and received monetary compensation or course-credits for their efforts. The setup, procedure and analyses were identical to those of Experiment 1, except for the details mentioned below.

#### Procedure

Experiment 2 consisted of two sessions, each performed on different days. Each session included two baseline blocks, a short practice and a grasping block, all following the same procedure as in Experiment 1. The main difference was the implemented trial sequence. We created a trial sequence with longer parts of 27 trials with a constant MD (*constant*), which were interrupted by short parts of nine trials with a pseudorandomized MD (*mixed*). The longer constant parts should allow building more stable predictions. The mixed interruptions were kept short to limit the total duration of the experiment allowing it to be completed in one session. Since we did not find an effect of the previous trial’s MD on tactile suppression in Experiment 1, we did not split the mixed sequence in *MD*_*same*_ and *MD*_*different*_ trials. Rather, we compared suppression only between mixed and constant parts. We presented three constant parts with a left MD and three blocks with a right MD, always interrupted by a mixed part. The sequence always started with a mixed part and consisted of 216 trials in total. The mixed parts were constructed in the same way as the mixed sequence in Experiment 1. Within the constant, longer parts, one of the two possible MDs was chosen, counterbalanced across participants, but the object position could still change within these parts. During the first session, participants were not informed about the structure of the presented sequence (*implicit version*). At the end of this session, participants filled out a custom questionnaire asking whether they observed a specific trial structure of the presented sequence. Only one of the twenty-four participants indicated that they noted some repetitions. During the second session, performed on average seventeen days later, the very same sequence was presented to each participant. During this session, the experimenter explicitly informed the participant verbally about the structure of the upcoming part of the sequence by indicating whether they would encounter a mixed or constant part (*explicit version*), without however indicating what mass distribution would be presented in the upcoming constant part.

#### Data analysis

Conditions were divided according to the parts of the sequence (mixed, constant) and the experimental version (implicit, explicit). It should be noted that in Experiment 2, we did not examine the trial-by-trial differences, but rather the differences that could emerge due to the overall context of repetitions or changes in MD. Particularly in the mixed condition, this is expected to result in a mean closer to zero for the digit separation index, which is comparable to calculating a mean for the values as outlined in Experiment 1. The effects of sequence part (mixed, constant) and experimental version (implicit, explicit) on digits’ separation, loading time, object roll, and tactile suppression were evaluated using separate 2 × 2 repeated measures ANOVAs. Significant interactions were inspected with post-hoc t-tests that were Bonferroni-corrected for multiple comparisons. Effect sizes are described as partial Eta squared (${\eta }_{p}^{2}$) for ANOVAs and as Cohen’s d for t-tests.

### Results

#### Kinematics

**Figure 5:**
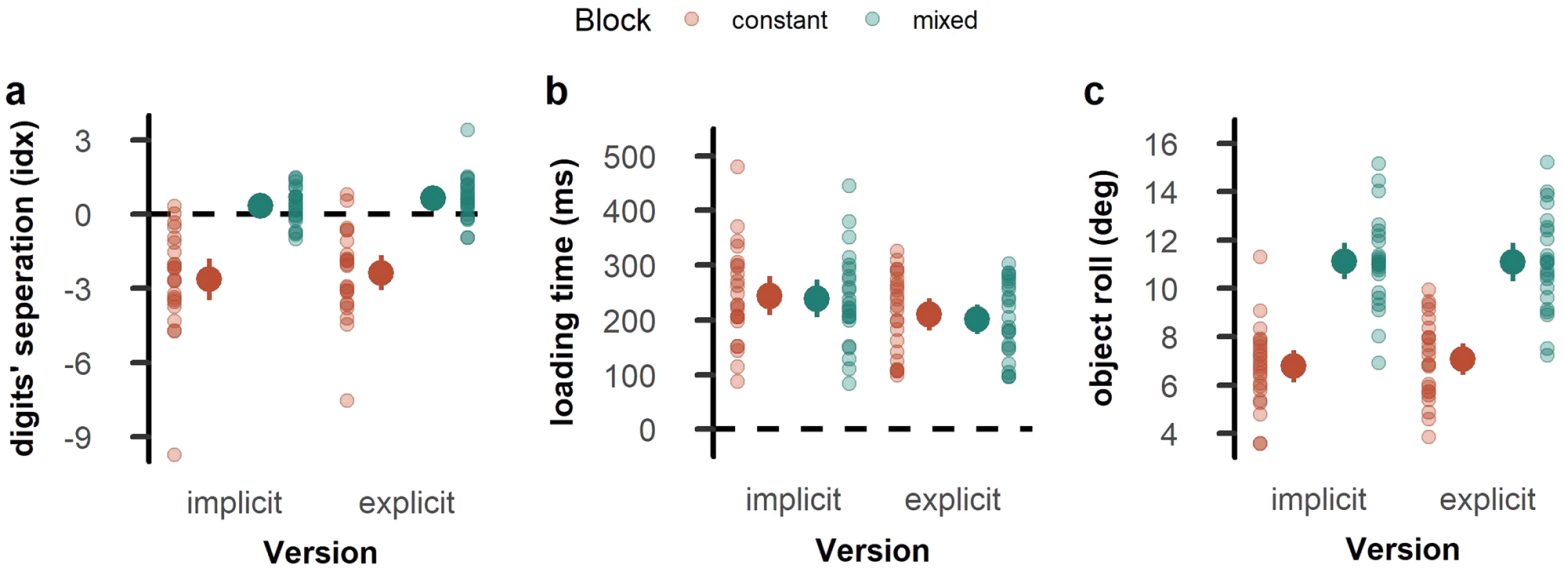
Kinematic performance in Experiment 2. (**a**) Digits’ vertical separation at the moment of object contact, (**b**) loading time, and (**c**) maximal object roll during the first 250 ms after object lift. The left side of each panel shows results for the implicit version, and the right side for the explicit version. Mean values for the constant blocks are depicted in red, those for the mixed blocks in green. Opaque dots represent the mean across participants with error bars indicating the confidence interval. Transparent dots represent individual participants.

For digits’ separation, a negative index occurring when the mass distribution is repeated and a positive when the mass distribution is switched, indicate that participants adopt in trial *n* a grasp configuration to match the dynamics experienced in trial *n* − 1, independently of whether the adopted grasping posture is suitable for the mass distribution of the grasped object. As expected, and in line with the results of Experiment 1, in the constant parts participants positioned their fingers in anticipation of a repeated MD in both the implicit (*t*_*23*_ = − 6.23, *p* < 0.001, *d* = − 1.27) and the explicit version of the experiment (*t*_*23*_ = − 6.53, *p* < 0.001, *d* = − 1.33). Likewise, in the mixed parts participants positioned their fingers in anticipation of a repeated MD, irrespective of whether the MD was repeated or changed. This was the case during both the implicit (*t*_*23*_ = 2.39, *p* = 0.026, *d* = 0.49) and the explicit version (*t*_*23*_ = 3.51, *p* = 0.002, *d* = 0.72). Evidently, these resulted in a main effect of sequence part (*F*_1, 23_ = 61.12, *p* < 0.001, ${\eta }_{p}^{2}$ = 0.73; Fig. 5a). We found no differences between implicit and explicit parts, nor an interaction (both *F* < 1.3, both *p* > 0.30, both ${\eta }_{p}^{2}$ < 0.05).

Participants took longer to start lifting the object when the MD was constant over several trials compared to when the MD changed (*F*_*1*,*23*_ = 6.82, *p* = 0.016, ${\eta }_{p}^{2}$ = 0.23). They also took longer to start lifting the object in the first, implicit version of the experiment, compared to the second, explicit version (*F*_*1*,*23*_ = 11.56, *p* = 0.002, ${\eta }_{p}^{2}$ = 0.33; Fig. 5b). There was no interaction between the two factors (*F*_1,23_ = 0.70, *p* = 0.411, ${\eta }_{p}^{2}$ = 0.02).

Object roll was smaller during the lift when the MD was repeated over several trials compared to when the MD changed (*F*_1,23_ = 470.69, *p* < 0.001, ${\eta }_{p}^{2}$ = 0.95; Fig. 5c), in line with the results of Experiment 1. There was no effect of the experimental version nor an interaction (both *F* < 2.5, both *p* > 0.13, both ${\eta }_{p}^{2}$ < 0.10).

#### Tactile sensitivity

**Figure 6:**
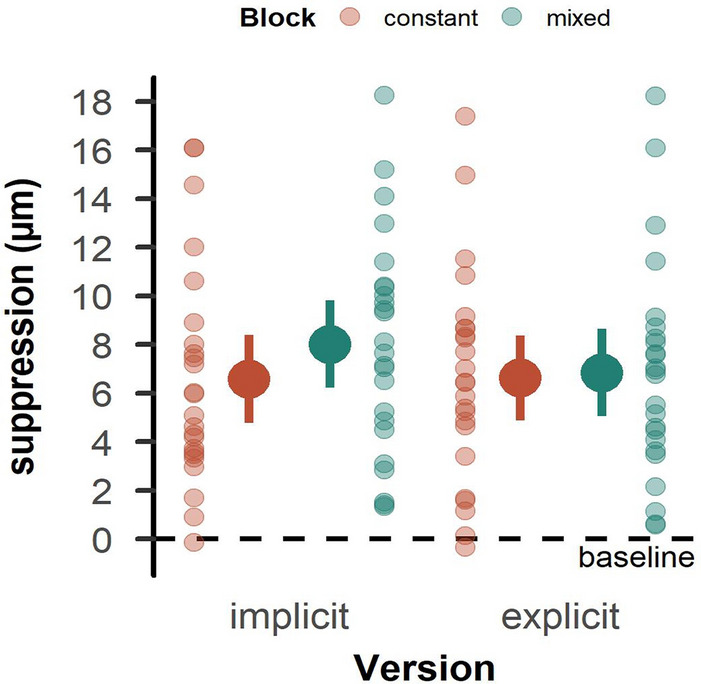
Tactile suppression scores in Experiment 2. The left side of each panel shows the implicit version, and the right side shows the explicit version. Mean values for the constant blocks are depicted in red, those for the mixed blocks in green. Zero values indicate no change from baseline, while higher values indicate a deterioration of tactile perception. Opaque dots represent the mean across participants with error bars indicating the confidence interval. Transparent dots represent individual participants.

Tactile suppression was evident during grasping compared to rest in the implicit version both in the constant (*t*_*23*_ = 7.15, *p* < 0.001, *d* = 1.46) and the mixed parts of the sequence (*t*_*23*_ = 8.72, *p* < 0.001, *d* = 1.78), as well as in the explicit version, again both in the constant (*t*_*23*_ = 7.41, *p* < 0.001, *d* = 1.51) and the mixed part of the sequence (*t*_*23*_ = 7.47, *p* < 0.001, *d* = 1.52). Suppression appeared stronger in the mixed than constant parts, but this difference was not statistically significant (*F*_*1, 23*_ = 4.01, *p* = 0.057, ${\eta }_{p}^{2}$ = 0.15). There was also no difference in suppression between implicit and explicit versions (*F*_*1, 23*_ = 0.45, *p* = 0.510, ${\eta }_{p}^{2}$ = 0.02; Fig. 6), indicating that the type of knowledge about the statistical regularities of the upcoming trial sequence did not systematically affect tactile suppression. There was also no interaction between the factors (*F*_*1, 23*_ = 2.69, *p* = 0.114, ${\eta }_{p}^{2}$ = 0.11).

### Discussion Experiment 2

In Experiment 2 we investigated whether longer parts of repeated object properties would lead to more reliable sensorimotor predictions and hence stronger tactile suppression. Further, we were interested in whether implicit or explicit information about the trial structure would affect tactile suppression. Grasping kinematic were affected similarly to Experiment 1, with grasping postures being predictively chosen to compensate for object roll, even if this prediction might have been wrong. We again found tactile suppression during grasping compared to rest, but suppression was similar between the constant and mixed parts. These results support and further extend the findings of Experiment 1, showing that sensorimotor memories influence movement kinematics but not associated tactile perception. The similar suppression between constant and mixed parts is, however, at odds with previous findings, where suppression was stronger when grasping an object whose mass distribution remained identical over trials than when it changed^6^. One possible explanation is that in the current study, participants were exposed to the constant parts for shorter durations and these constant parts were alternating with mixed parts, which might have limited the establishment of reliable predictions that could affect tactile suppression.

Implicit and explicit information about the upcoming trial structure did not affect the grasping posture or object roll, and it had no effect on tactile suppression. Yet, our results showed that during the explicit session of the experiment, participants started lifting the object earlier than during the implicit session. We interpret this finding with caution, as the explicit version was presented always *after* the implicit version. This does not allow us to conclude whether this effect is caused by the explicit information itself or whether it is a byproduct of practice, as participants may have lifted the object faster simply because they were more familiar with the task.

## General discussion

In this study we examined whether sensorimotor memories influence grasping kinematics and associated tactile processing. Participants reached to grasp an object that could have one of two possible mass distributions. When the mass distributions were presented in a pseudorandom order across trials (Experiment 1), participants grasped the object in the second of two consecutive trials as if it had the mass distribution experienced in the previous trial, demonstrating predictive grasping behavior based on sensorimotor memories. Similar behavior was observed in Experiment 2, when the mass distributions were presented both in a mixed and in a constant order. To assess tactile processing, we employed the well-established phenomenon of tactile suppression and found clear suppression of tactile signals during grasping compared to rest. However, the strength of tactile suppression was unaffected by the statistical regularities of the trial sequence. Our results indicate that sensorimotor memories exert a strong influence on movement behavior but not on associated tactile processing.

Goal-directed behavior is primarily based on visual information. For instance, when grasping an object, humans use vision to extract relevant object properties, such as its size^1^, orientation^2^, shape^3^, or surface material^4^, and they choose suitable grasping points already during movement planning^30, 31^. However, visual information is not always available or informative about certain object properties. For instance, the object’s center of mass may not always be inferred from vision. When interacting with such objects, humans initially adopt a ‘default’ motor behavior, and if object properties remain invariant and occur recurrently, they use somatosensory feedback from the previous interaction to predict the object properties and tailor their movement accordingly^7^. However, when object properties change continuously, predictions are less reliable and can be misleading. Yet, even in such cases, humans plan their movements assuming that the object in question has the same properties as those experienced in the previous trial^24, 25, 32^. Our experiments support such predictive behavior. Specifically, participants grasped the object in the second of two consecutive trials as if it had the dynamics experienced in the trial before. Here, the grasping posture cannot have been chosen based on somatosensory feedback from the object of the current trial because we measured this grasping posture before such feedback could be utilized. This tuning of grasping posture demonstrates that motor plans are established based on sensorimotor memories. Evidently, such memories can be advantageous when the prediction is correct but disadvantageous when it is incorrect, as reflected in object roll during object lift.

The second aim of the study was to examine whether tactile processing associated with object grasping and manipulation is affected by predictions established through sensorimotor memories. To capture the original prediction about the object dynamics based on the object’s behavior in the previous trial and to examine its influence on tactile processing, we stimulated at the moment of first contact with the object, just before participants lifted it and received sensory information about its mass distribution. It has been shown that tactile suppression is stronger in the early than late phases of a grasping movement suggesting a greater dependence on predictive control during this period^10, 33, 34^. The results of our two experiments demonstrate robust tactile suppression during movement, in line with previous findings^6, 10^. This supports the idea that sensorimotor predictions established during movement reduce associated tactile sensitivity^14^. In Experiment 1 we show that tactile suppression did not differ between trials of the same than different mass distribution compared to the distribution experienced in the previous trial. This suggests that, although predictions from sensorimotor memories influence grasping kinematics, these predictions do not affect tactile suppression. One possible explanation for this finding is that we assessed tactile suppression at the moment of first contact with the object. At that moment, participants had no access to sensory input about the actual mass distribution. This guaranteed that the measured tactile suppression was influenced only, or at least primarily, from the established sensorimotor predictions, without additional effects of the instantaneous sensory input. However, this experimental decision may also mean that the sensorimotor prediction at that moment might have been equally strong independently of the object’s actual mass distribution. In such case, it is unsurprising that tactile suppression is similar between conditions. In Experiment 2 we included a condition with sequences of 27 consecutive trials that involved an object of constant mass distribution. Such constant parts should facilitate predictions about the object’s mass distribution compared to the mixed sequence, in which the mass distribution was changing on a trial-by-trial basis. Despite the long sequences of trial repetitions in the constant parts, tactile suppression was similar between constant and mixed parts. This is at odds with previous findings that demonstrated stronger suppression in constant than mixed sequence parts^6^. Such difference may arise due to the fact that in our current experiment the mixed and constant parts were relatively short and were presented interchangeably, whereas in the other study the mixed and constant parts were much longer and were presented in distinct blocks. Thus, in the present study, tactile suppression might not have been adapted as effectively, as participants had to constantly adjust to a switch between constant and mixed intercepts, and as a consequence the sensorimotor predictions in the constant parts might have been weaker compared to the previous study^6^. Future research could explore how tactile sensitivity changes during the grasp and lift movements and how it is affected by predictive violation with different stimulation times throughout the course of the movement.

Tactile suppression has also been explained by peripheral mechanisms, such as backward masking from movement-related afferent signals that mask the tactile probe used to measure suppression^19^. In this case, we would have expected stronger suppression in trials where the prediction about the object’s mass distribution was wrong, such as in *MD*_*different*_ than *MD*_*same*_ trials of Experiment 1. This is because, when experiencing an unpredictable sensory event during goal-directed actions, sensory feedback gains increase^28^, and so such an increase in feedback processing might mask the previously presented tactile probe stimulus. In Experiment 2, it appears that suppression is qualitatively stronger in the mixed compared to the constant condition of the implicit version, however this was not systematic. All in all, our results do not lend support for the hypothesis of backward masking either, and the apparent invariance in suppression as a function of feedback signals is in line with previous findings^20^.

In Experiment 2 we further examined whether grasping kinematics and tactile suppression are sensitive to statistical regularities that are implicitly or explicitly inferred. Previous research has demonstrated that implicit learning of the underlying dynamics can be beneficial for sampling, for instance through haptic exploration^35^. In addition, implicit sensorimotor memories of object properties can influence the anticipation of these properties^36^, both in the absence and presence of explicit cues^37^. Yet our kinematic results did not reveal any benefit from the explicit instruction about the trial sequence. Albeit, we observed a slight decrease of loading times when explicit information about the upcoming sequence was provided. However, it is noteworthy that this benefit may simply be due to the repetitive performance of the task, as the explicit session was *always* presented after the implicit session, so we cannot disentangle effects of practice from effects of the type of information. Importantly, we also did not observe any effects of implicit or explicit information on tactile suppression. Informing participants about the fact that the upcoming trials would be of a mixed or a constant sequence does not have any strong effect on motor behavior or associated tactile processing.

In conclusion, we demonstrate that predictions based on sensorimotor memories influence grasping kinematic by adjusting the grip to the previously experienced object properties. However, associated tactile suppression appears robust to such previous trial effects.

## Data Availability

The data collected for this work will be publicly available at https://doi.org/10.17605/osf.io/w6ruy.
